# Tropical decadal variability in nutrient supply and phytoplankton community in the Central Equatorial Pacific during the late Holocene

**DOI:** 10.1038/s41598-024-54635-3

**Published:** 2024-02-20

**Authors:** T. P. Guilderson, D. S. Glynn, M. D. McCarthy

**Affiliations:** Ocean Sciences Department, University of California – Santa Cruz, 1156 High Street, Santa Cruz, CA USA

**Keywords:** Palaeoceanography, Palaeoclimate, Marine chemistry, Geochemistry

## Abstract

We have reconstructed baseline δ^15^N and δ^13^C of export production at Kingman Reef in the Central Equatorial Pacific (CEP) at sub-decadal resolution, nearly continuously over the last 2000 years. The changes in δ^15^N reflects the strength of the North Equatorial Counter Current (NECC) relative to the South Equatorial Current (SEC), and to a lesser extent, the North Equatorial Current (NEC). Seasonal to multi-decadal variation in the strength of these currents, through the redistribution of heat, have global climate impacts and influence marine and terrestrial ecosystems. We use modern El Niño-La Nina dynamics and the Tropical Pacific Decadal Variability (TPDV) pattern, which is defined in the CEP, as a framework for analyzing the isotopic data. The CEP δ^15^N and δ^13^C records exhibit multi-decadal (50–60 year) variability consistent with TPDV. A large multi-centennial feature in the CEP δ^15^N data, within age-model uncertainties, is consistent with one of the prolonged dry-pluvial sequences in the American west at the end of the Medieval Climate Anomaly, where low TPDV is correlated with drier conditions. This unique record shows that the strength of the NECC, as reflected in baseline δ^15^N and δ^13^C, has at quasi-predictable intervals throughout the late Holocene, toggled the phytoplankton community between prokaryotes and picoplankton versus eukaryotes.

## Introduction

The redistribution of excess incoming solar radiation (energy) in the tropics by the atmosphere and ocean dictates much of Earth’s climate system. The redistribution of energy as latent and sensible heat drives weather patterns which impacts billions of people, as well as strongly influencing terrestrial and marine ecosystems. Variability in sea surface temperature, which impacts weather and ultimately climate, occurs at sub-seasonal to multi-decadal scales. The tropical Pacific decadal sea surface temperature (SST) anomaly pattern (TPDV) has its highest amplitude in the Central Equatorial Pacific (CEP) and is similar to the pattern of El Niño-Southern Oscillation (ENSO) SST anomalies^1^. Decades with more La Niña events and fewer El Niño events have lower TPDV and the converse is also true. Interannual (ENSO) and decadal variability (TPDV) reflects and impacts the export of latent and sensible heat from the tropics to extra-tropics which sets up major weather patterns through the ascending and descending limbs of the Hadley Circulation and the position of the jet stream. TPDV is somewhat analogous to the Interdecadal Pacific Oscillation (IPO)^2^, and the Pacific Decadal Oscillation^3^. These SST patterns are dynamically connected to each other through tropical-extratropical teleconnections which nearly synchronize the north and south subtropical Pacific^4^. Together the TPDV and IPO explain a significant portion of global SST variability^5^. Modulations of the strength, intensity, and pattern of decadal SST (and sea level pressure) variability not only strongly impacts weather and weather extremes but marine ecosystems^3,6,7^.

By itself the instrumental record is not long enough nor with appropriate geographic coverage to record more than a few multi-decadal oscillations. Due to the lack of records with sufficient fidelity to capture multi-decadal variability the timing and drivers of oscillations before this period are poorly understood. Questions exist on how external forcing (e.g., greenhouse gases and aerosols from the industrial revolution or volcanoes) has influenced the pattern of decadal variability^8^, and if the variability is intrinsic to the ocean–atmosphere system as an internal periodic oscillation or whether it is stochastically forced quasi-periodic variability^9,10^. This leads to the question whether or not the recent past is a ‘clean’ analog of the instrumental era^11,12^. A related question is partitioning natural climate variability, as climate warmed out of the Little Ice Age (LIA) and internal multi-decadal variability from that of anthropogenic induced change^13^. Paleoclimate reconstructions, particularly those with seasonal to sub-decadal resolution (e.g., tree-rings, reef-building corals, speleothems, some sediment records), gap-fill in time and geography. Multi-decadal variability that is likely related to TPDV/IPO/PDO has been documented in paleo-records from, for example: sediments of the Santa Barbara Basin^14^, in speleothems that are influenced by the SE Asian and Australian monsoon^15–17^, speleothems that capture the South Pacific Convergence Zone^18,19^, and in tree-ring drought records from North America^20,21^ and SE Asia^22^. To date, however, there are no continuous multi-millennial sub-decadal resolved records from the CEP. This is the core region of the TPDV, and paleo-records are needed to answer if the variability is an intrinsic aspect of Earth’s climate system or if it has changed over longer (geological) timescales with, for example, different insolation patterns or other external forcings.

The Line Islands, a series of atolls and coral islands straddling the equator in the Central Pacific, are uniquely situated to elucidate past variations in sea surface temperature, salinity^23,24^ and rainfall associated with ENSO and/or the migration of the inter-tropical convergence zone (ITCZ)^25–27^. At 6° N, in the equatorial waveguide, Kingman Reef and nearby Palmyra Atoll are generally bathed by waters of the eastward flowing North Equatorial Counter Current (NECC)^28–31^. The NECC is sourced in the western equatorial Pacific from the Mindanao Current, and to a lesser extent the North Equatorial Current, which bring North Pacific Gyre surface water into the low-latitude tropics^32^. Interannual variations in the strength of NECC transport are associated with ENSO, and have the ability to bring significant amounts of heat from the western equatorial Pacific to the central and eastern Pacific during El Niño events^28,30,33^. During El Niños when the equatorial trade winds slacken or reverse, water from the North Pacific Gyre expands into the western equatorial Pacific^34^. To a large extent, SST and nutrients of surface water masses which mix in the CEP co-vary.

**Figure 1:**
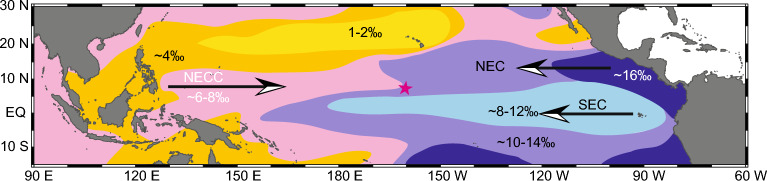
Estimated surface ocean (0–130 m) δ^15^NO_3_ in an ocean general circulation model. Arrow vectors denote the main position of the equatorial branch of the South Equatorial Current (SEC), the North Equatorial Current (NEC), and the North Equatorial Counter Current (NECC). Star denotes the position of Kingman Reef and nearby Palmyra Atoll. The surface δ^15^NO_3_representation is intended to show the general patterns of the east to west and north to south δ^15^NO_3_ gradients, and has been adapted from Somes^91^. Although some of the details are different, particularly the westward extent of the NEC’s δ^15^NO_3_ signature, the model output is sufficiently similar to an observation-based interpolation^92^ for framing that the western equatorial Pacific has lower δ^15^NO_3_ compared to waters advected from the east, and that waters which feed the NECC are primarily derived from the North Pacific Subtropical Gyre.

The source waters to the Line Islands from these different regions have highly distinct nitrate (NO_3_) isotope values. NECC nitrate levels in the western Pacific are low, often below detection limits, and comparable to the subtropical gyres^35^. In the western equatorial warm pool and the subtropical gyre nitrogen fixation augments intrinsic nitrate leading to low δ^15^NO_3_ values (0–5‰). Near the equator rates of nitrogen fixation decrease to the east^36–38^. Conversely, in the eastern Pacific, the NEC and SEC both have higher nitrate concentrations and more positive δ^15^NO_3_ values due to advection of waters impacted by denitrification signatures, as well as the fact that residual nitrate which has been upwelled to the surface has undergone progressive Rayleigh fractionation associated with incomplete nutrient utilization as waters are advected away from upwelling locations^39–41^. Consequently, surface δ^15^N-nitrate values at the equator are very high, δ^15^N values of 13–18‰ between 155° W and 170° W, before values decline northward into the subtropical gyre^39,41^. Nitrate isotope values of source waters of the east and west Pacific do not appear to have substantially shifted over the late Holocene^42–45^. This sets up a natural water-mass nutrient and ^15^N-isotope gradient which can be exploited to reconstruct water-mass variability in the CEP (Fig. 1, see also Supplementary Fig. 1).

Deep-sea proteinaceous corals are one of the best bioarchives to reconstruct surface water source and chemistry variations. *Kulamanamana haumeaae*, a deep-sea colonial zoanthid, is a low-order consumer^46^, and like other deep-sea corals, feeds primarily on recently exported sinking particulate organic carbon which tracks the carbon and nitrogen isotope values of sinking particulate organic carbon, i.e., export production^47^. The polyps create a hard, horny protein (gorgonin) skeleton, nearly purely amino acid based, which is resistant to degradation over multi-millennial timescales^48,49^. These amino acids are from surface production: either directly (source and essential amino acids) from diet or indirectly from diet via biosynthetic synthesis (trophic and non-essential). Bulk proteinaceous coral skeletal isotope values (δ^13^C, δ^15^N) are strongly correlated with source (δ^15^N) and essential (δ^13^C) amino acid values^47,48,50^, and provide insight into baseline nitrate variability and the impact of phytoplankton community structure on export production. Here, we present sub-decadal resolved reconstructions, spanning the last 2000 years from Kingman Reef (6° N, 162° W) in the Line Islands complex in the CEP, reflecting the isotopic composition of baseline nitrate (NO_3_) in surface waters supporting phytoplankton and export production. The changes in δ^15^N reflects the strength of the North Equatorial Counter Current (NECC) relative to the South Equatorial Current (SEC) and to a lesser extent, the North Equatorial Current (NEC). Corresponding carbon isotope data likely track changes in extant plankton assemblages, in particular variations between diatom and eukaryotic populations versus prokaryotic and/or nitrogen fixing algae. While our records do not resolve individual ENSO events, we will use modern ENSO dynamics as a framework for analyzing past changes.

## Results and discussion

**Figure 2:**
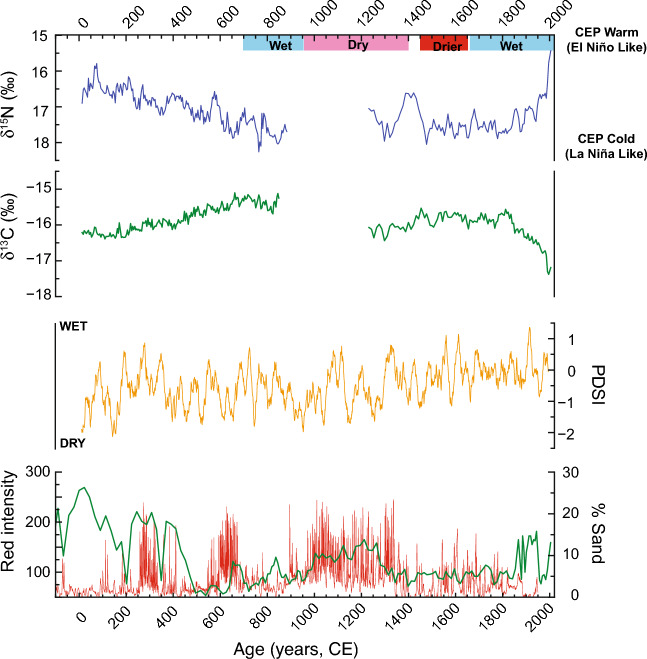
Comparison of CEP TPDV as reflected by δ^15^N (blue line, this study), and δ^13^C (green line, this study), American West Palmer Drought Severity Index for the North American west (33–43° N, 106–119° W: data of Cook^20,21^), and two eastern Pacific El Niño proxy records: El Junco (1° S, 89° W, 700 m asl) of San Cristobal Island in the Galapagos Archipelago %Sand Index (green line, left axis: data of Conroy^60^) and the red color of sediment laminae in Laguna Palcacocha (2.8° S, 79.2° W, 4060 m asl) as reported in Moy^61^. The El Junco record has an age uncertainty that ranges from ± 10 years to ± 195 years and the Laguna Palcacocha may be biased < 100 years (IntCal98 versus SHCal20 framework). More (less) positive δ^15^N values are interpreted to reflect La Nina-like (El Niño-like) conditions and lower (higher) TPDV. In general, more positive δ^13^C values are interpreted to reflect a phytoplankton community dominated by eukaryotes including diatoms whereas less positive δ^13^C values reflect prokaryote and pico-plankton dominated communities. Washington Lake on Teraina Island (4.7° N, 160.4° W, near sea level) wet and dry periods as recorded in plant wax lipid biomarkers and sediment physical properties are noted. When Lake Washington dries out early in the LIA, locally the ITCZ is thought to be south of Teraina and thus south of Kingman Reef and Palmyra Atoll^25–27^. The large, multi-centennial TPDV excursion at the beginning of K1 (1200–1500) is, within age-model uncertainties, coincident with the American West and Sierra megadrought—pluvial-megadrought sequence noted by Stine^62^ and recorded in tree-ring reconstructions of regional PDSI.

Collectively, the live-collected and sub-fossil *Kulamanamana* specimens capture, nearly continuously, the last 2000 years (Fig. 2). Calibrated age model uncertainties average ± 110 years (95% confidence interval) with the live-collected specimen (2005–1222 CE) having a resolution of 7 years per sample and the sub-fossil specimen (922–13 CE) approximately 5 years per sample. Diagnostics: amino acid mole percent (AA mol%) and carbon:nitrogen ratios (C/N) indicate good preservation of both skeletons and that except for the outermost 1–2 mm of the sub-fossil skeleton, the bulk stable isotope data are not compromised by diagenesis^45,49^. These specimens’ skeletal compound specific isotope analysis of amino acids (CSIA-AA) nitrogen data show a strong relationship between skeletal bulk and source AA δ^15^N values, allowing us to interpret bulk values as primarily tracking baseline δ^15^NO_3_ (Supplementary File 1).

Across the full data-set, the δ^15^N reconstruction exhibits complex behavior (Fig. 2). We document a long secular multi-centennial increase (~ 75–850 CE), a multi-centennial scale excursion during the end of the Medieval Climate Anomaly (MCA), a post Little Ice Age (LIA) rapid or sharp decrease (1860–2005 CE), and multi-decadal variability in both specimens. Except for the post- LIA and into the modern Industrial Era and its Suess effect^51^, δ^13^C variability exceeds that predicted from changes in atmospheric δ^13^C as reconstructed in ice cores^52^ or the impact of temperature on carbon isotope systematics in plankton^53^. In general, δ^15^N and δ^13^C values inversely covary, although the carbon isotope amplitude is smaller than that for nitrogen. For all the pre-industrial data 38% of the variance is shared with an evolution of tighter coupling in the older portion of the record. The only exception is the post-LIA and industrial era, in the younger portion of the record (see Supplementary File 1).

Within the ENSO model framework more positive δ^15^N indicates more frequent if not stronger La Niña events, a cooler CEP and lower TPDV. In contrast, less positive δ^15^N indicates more frequent/stronger El Niño events, warmer SSTs, and higher TPDV. Local impacts influencing baseline nitrate go hand in hand with the ENSO framework and amplify the baseline δ^15^N response. Local impacts such as time spans of increased stratification due to warmer temperatures and/or lower salinity due to increased local precipitation, could promote a local increase in nitrogen fixation. Such local effects tend to occur during El Niño events and a stronger influence of the NECC^31^. Through atmospheric teleconnections, a cooler CEP with low TPDV has been shown in climate models to lead toward a drier western North America^54^, which is similar to ENSO teleconnection impacts on interannual timescales.

Across the Late Holocene at the Peru Margin, the source of the SEC, sedimentary bulk δ^15^N has less than half a per mil range, or constant when considering analytical uncertainty (see Supplementary File 1). There is a similar lack of variability in open ocean cores from the eastern tropical Pacific^44,45^. We also do not observe significant variability in sediment records from the region where the NECC originates (see Supplementary File). Thus, the endmembers of δ^15^NO_3_ values in the two main water masses which influence our site have not significantly changed prior to the end of the LIA and into the industrial era.

**Figure 3:**
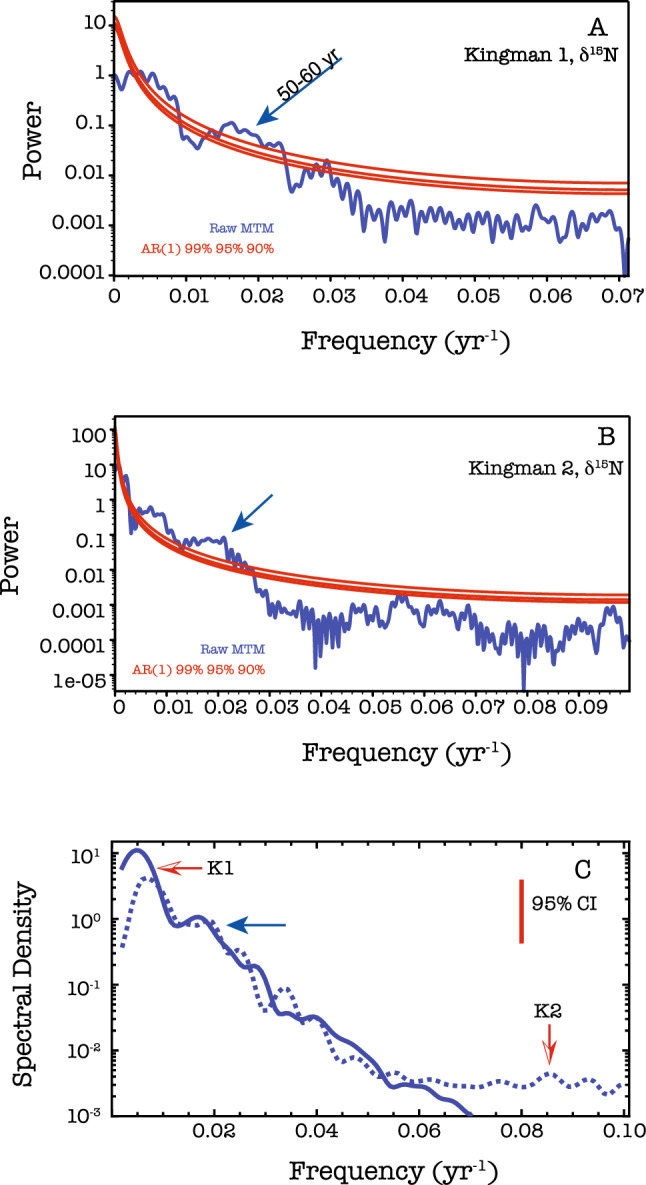
Spectral analysis of the Kingman Reef δ^15^N data as a function of frequency (year^−1^). (**A**) Multi-taper method (MTM) results of the K1 specimen (1220–1880 CE). (**B**) Multi-taper method results of the K2 specimen (13–858 CE) and (**C**) Blackman–Tukey results of K1 (solid, bold line) and K2 (dashed line), together. Confidence significance levels for the MTM results were determined using robust red-noise intrinsic to the data. The 95% confidence interval is shown for the Blackman–Tukey results. Data were handled as described in the "Methods" section. The additional arrow highlights the 50–60 year period, consistent with our understanding of TPDV. For clarity, the announcement of the 50–60 year period is only in (**A**).

We posit, therefore, that our CEP δ^15^N record is primarily capable at capturing the frequency of the mixing of source waters in the CEP, as opposed to the ability to precisely apportion the fraction of eastern versus western water. This is because, and although the δ^15^NO_3_ NECC, SEC, and NEC source endmembers do not appear to have significantly changed over the last 2000 years, that nitrate uptake by phytoplankton in SEC and NEC waters which are advected westward modifies the residual NO_3_ along a Rayleigh fractionation pathway leading towards more positive δ^15^NO_3_ values^55^. Thus, the ultimate δ^15^NO_3_ value of waters which are entrained into the NECC, or via the SEC directly impinging upon the northern Line Islands during sustained periods of anomalously strong trade winds, is driven by a complicated convolution of productivity changes. Such changes are very likely associated not only with trade-wind strength but also iron availability: through upwelling and entrainment of the Equatorial Under Current and Subantarctic waters^56,57^, dust deposition^58^, and recycling of iron^59^. Additionally, if in the far western Pacific the NECC endmember δ^15^NO_3_ changes due to nearly basin-wide changes in δ^15^NO_3_, it mainly would change the amplitude of the CEP δ^15^N variability but leave the spectral character intact, because the general feature of less positive values in the NECC and more positive in the SEC and NEC would remain. Our NECC TPDV δ^15^N proxy record has its highest multi-decadal variability at 50–65 years (Fig. 3). This frequency range is similar to speleothem based reconstructions of the SPCZ^18,19^ and the SE Asian and Indo-Pacific monsoon systems^15–17^.

Eastern Pacific proxy reconstructions of ‘classic’ El Niño^60,61^ indicate that the early part of our record, ~ 0–500 (Common Era) CE, is replete with stronger and or more frequent El Niños. Around 500 CE ENSO transitions into more frequent La Niñas (Fig. 2). Our CEP δ^15^N record, in addition to visually striking, and statistically significant multi-decadal variability, captures this shift in tendency as the trend towards more positive δ^15^N values indicative of a stronger influence of SEC water. Furthermore, the visual correlations between CEP δ^15^N and the US American West Palmer Drought Severity Index (PDSI) record are intriguing, albeit we note that with our age-model uncertainties there are nearly as many instances of a mis-match at the < 100 year level as there are matches. The largest multi-centennial feature in the CEP δ^15^N data, within age-model uncertainties, is consistent with one of the prolonged dry-pluvial sequences in the American west at the end of the MCA^20,21,62^, i.e., the pluvial between Stine’s two massive droughts. We do not have complete coverage through the MCA to ascertain the decadal variability in the CEP. The observed data and correlations, however, suggest that the CEP was likely in a state of more frequent and possible intense La Niña events with low TPDV leading to a cooler CEP. This is similar to correlations over ENSO timescales. Over their common overlap in time, our data and the tree-ring based North American PDSI reconstruction share common multi-decadal variability (see Supplementary File).

During this time interval, ~ 0 to 700 CE, a speleothem-based reconstruction of the Indo-Australian and SE Asian monsoon systems^15^ also has what has been interpreted as a decrease in the intensity of monsoon rainfall and a contraction of these systems. Although contrary to a different putative ITCZ reconstruction which includes records across the whole of the tropics^17^, this is consistent with our record of more La Niña like character, stronger trade-winds, and reduced TPDV. Leaf-wax deuterium isotopes^63^, a rainfall proxy, from Indonesia implies a stronger Indonesian Monsoon across 0–1600 CE punctuated by periods of weaker monsoon intensity during − 100 to 320 CE (± 200 years, 95%CI) ~ 850 to 1230 (± 200 years, 95% CI). The latter period of weaker Indonesian Monsoon is replicated in speleothem-based data from Thailand^16^. However, the inter-tropical convergence zone (ITCZ) is not a monolith and is separate from the terrestrial monsoon systems which are more directly anchored to landmasses. Thus, we may anticipate periods when the pan-Pacific or local CEP position of the ITCZ does not exactly reflect the inferred position and intensity of the monsoon-based systems.

Entering the Little Ice Age period, biomarker and sediment physical property reconstructions of the mean position of the ITCZ indicates that for a portion of the LIA, approximately 1420 to 1600 CE, locally the ITCZ was south of Washington Island, which is at 4.7° N within the Line Islands^25–27^ and thus south of Kingman Reef. Baseline δ^15^NO_3_ values through this period are consistently less positive than earlier in the record and exhibit smaller amplitude multi-decadal variability. Low amplitude δ^15^N variability is a characteristic of the LIA, even when the ITCZ moves back north of Washington Island. Low TPDV and a cool CEP is consistent with proxies of ENSO where El Niños are less frequent or intense. This is however separate to the inference of increasing precipitation over the Galapagos Islands in the eastern Pacific, even in the absence of frequent or strong El Niños^60,64^. Within the LIA, Indonesian and SE Asian monsoon proxies generally include more intense and less intense centennial scale periods^16,22,63^.

The modern, post-LIA and industrial era δ^15^N exhibits both multi-decadal variability and a large, nearly 2‰, decrease in values. The rate of change during the latter half of the twentieth century exceeds that of the centennial scale excursion in the MCA by a factor of two to three (0.3‰-decade^−1^ vs. ~ 0.1‰-decade^−1^), with comparable rates over 100s of years. Reconstructions of the δ^15^N in the Mindanao Current from sediment cores^43,44^ do not have the fidelity to capture this decrease. After the 1976/77 shift in the IPO/PDO, the tropical Pacific experienced multiple strong El Niño events, which in our ENSO framework would lead to a stronger NECC bringing warm, low δ^15^NO_3_ water to the CEP. Because of its length, the CEP δ^15^N expression may miss the shift back to weaker El Niño events post 2000^65^ and thus, the greater impact of cooler more positive δ^15^NO_3_ water in the CEP. Missing half of the oscillation would give the appearance of a massive secular δ^15^NO_3_ shift. A modification to this interpretation is that there is indeed a change in the δ^15^NO_3_ in the western Pacific associated with a post-LIA/industrial era increase in nitrogen fixation. The increase in nitrogen fixation could be specific to the Western Pacific or it could include the North Pacific surface gyre waters from which the NECC is sourced.

Near the Hawaiian Islands in the North Pacific Subtropical Gyre, post-LIA and through the industrial era baseline δ^15^NO_3_ decreased due to a phytoplankton community with more nitrogen fixing bacteria in response to increased stratification^48,50^. Within the gyre, the Hawaiian Islands span a natural south to north less oligotrophic to more oligotrophic gradient^48^. The post LIA δ^15^N amplitude of change, 1.2–1.4‰, near French Frigate Shoals is less than near Oahu (~ 2‰) and Cross Seamount (~ 2.5‰). Based on estimates of nitrate not supported by Redfield ratio remineralization, N*^66^, French Frigate Shoals may be more representative of the gyre as a whole. Gyre surface water expanding into the western Equatorial Pacific during El Niño events and an increase in entrainment of gyre surface waters with low δ^15^N values into the NEC would, in tandem, work to produce the observed CEP δ^15^N trend. Such an interpretation would also explain twentieth century long-term δ^15^N variability at the base of the nitricline in the Western Pacific^67,68^. These data imply that similarly to the NPSG, regionally in the CEP, due to increased stratification coincident with more frequent El Niño events, has also seen an increase in nitrogen fixation and an implicit change in the phytoplankton community post LIA.

The δ^13^C records, while not having as large amplitude changes, are largely consistent with δ^15^N data within our interpretational framework. In the δ^13^C time-series, excluding the post-LIA/industrialized era, there are three main long-term δ^13^C value changes: increase between ~ 200–700 CE and ~ 1250–1500 CE during the MCA, and an approximate plateau from ~ 1500 to 1820 CE during the LIA. All these δ^13^C changes exceed those of atmospheric CO_2_^52^ and thus cannot be solely the result of air-sea δ^13^CO_2_ exchange and equilibration. Post-LIA and into the industrialized era the near continuous 1.2–1.5‰ δ^13^C decrease is ~ 60% of the decrease observed in the atmosphere and interpreted to be dominated by the Suess Effect^51^. The coupling between δ^13^C and δ^15^N, i.e., a tendency towards commensurate directional changes in carbon and nitrogen isotope values, is reflected in the similarity of the δ^13^C data’s spectral analysis (Supplementary Fig. 9). Like δ^15^N, the δ^13^C data exhibit consistent multi-decadal variability with 50–60 year periodicity.

We interpret the pre-industrial era δ^13^C data in a phytoplankton community framework. CSIA-AA δ^13^C in live-collected and sub-fossil *Kulamanamana haumeaae* from off Oahu have confirmed the ability of bulk skeletal carbon isotopes in *Kulamanamana* to capture broad changes in community structure^46,50^. Different main phytoplankton groups have ranges of isotopic fractionation broadly linked to cell size, growth rate, and other factors, relative to in situ dissolved inorganic carbon^53,69,70^. Major groups of the equatorial Pacific include Prochlorococcus, Synechococcus, nano-eukaryotes and diatoms. Picoplankton typically have more negative δ^13^C values compared to larger phytoplankton species. Diatoms, in particular, have characteristically enriched δ^13^C values linked to cell size and often faster growth rate in nutrient replete conditions^69–73^. In general, the relative abundance of these taxons leads to more positive δ^13^C primary and export production values near the equator and more negative values in the oligotrophic gyres^73^.

In the NPSG this framework can not only explain most δ^13^C shifts, but also explains coupled δ^13^C and δ^15^N changes^50^. Skeletal δ^15^N values are generally coupled with δ^13^C values in both records (K1 r^2^ = 0.55, K2 r^2^ = 0.65), consistent with the expectation that coupled atmosphere–ocean dynamic changes associated with the strength of the NECC toggles community composition between a community of prokaryotes and picoplankton (lower δ^13^C values) versus eukaryotes such as diatoms (more positive δ^13^C values). Specifically, the periods of lower δ^13^C values (during 0–200 CE, ~ 1200–1400 CE) suggest greater abundance of smaller celled prokaryotes, coincident with weakened trade winds, and a stronger NECC. Periods of higher δ^13^C (e.g., during ~ 500–850 CE and 1450–1850 CE), in contrast, represent enhanced larger cell (especially diatom) contributions, which would be expected to be facilitated by increased nutrient transport from the east Pacific during La Niña-like conditions.

There are also some exceptions to the typical tight coupling of δ^13^C and δ^15^N values in the record. The clearest exceptions are the ~ 1‰ oscillations in δ^15^N values found in the K2 record and the first half of the multi-centennial δ^15^N excursion ~ 1350 to 1500 CE in the K1 record. We hypothesize that oscillation events observable in δ^15^N during these decoupled periods may be tracking changes in source water δ^15^N signatures, rather than localized changes in stratification and nitrate concentration which would be expected to influence the overall phytoplankton community, and so also δ^13^C values. We note that similar “decoupling” behavior between δ^13^C and δ^15^N has been documented in other sub-decadal resolved marine isotope records over the last 5000 years^45^. One possible interpretation of the decoupling between δ^13^C and δ^15^N which has previously been proposed is a shift in phytoplankton community composition between nitrate utilizing cyanobacteria versus nitrogen fixing cyanobacteria with a less positive δ^15^N signature. This would be expected to alter δ^15^N of export production without substantially changing δ^13^C values as the phytoplankton composition remains prokaryotic^50^.

## Conclusions

We have determined that TPDV, as reflected by water-mass dynamics in the CEP, has been a near constant fixture in the climate system over at least the last 2000 years. There is at least one period of centennial scale low TPDV which, based on modern teleconnections, is consistent with extreme drought in the American Southwest. We hypothesize that there is a second significant centennial scale period of low TPDV in the data gap between our two individual records. Due to age-model uncertainties in our record we cannot yet clearly determine if the CEP TPDV has a tight, narrow periodicity which could imply an internal oscillation intrinsic to the coupled climate system. Moreover, better chronological control is required to refine the visual correlations we have made with ENSO and drought proxies and to ascertain if there are slight temporal offsets between land-mass monsoon and drought proxies and open ocean CEP sites influenced by the ITCZ. This is regardless of the commonality in the frequency domain of monsoon proxy and terrestrial drought records which exhibit similar multi-decadal variability. Through atmospheric teleconnections CEP variability is expressed in regional drought and pluvial extremes. The general coupling between δ^13^C and δ^15^N that we observe in our records is consistent with the framework where physical current (ocean dynamics) and climate regime directly influence phytoplankton community across timescales. In our framework, generally, times of low TPDV promote eukaryote-based phytoplankton communities whereas during high TPDV prokaryote and pico-plankton-based communities, including nitrogen fixers, are selected for.

The centennial period of low TPDV coincident with North American drought provides a natural testbench for models to elucidate not only how TPDV varies with background climate but how the background climate state influences atmospheric teleconnections in models. Modelling studies of future tropical Pacific decadal variability have a wide range of results^1,5^. Thus, our data provide a new benchmark for estimating, through hindcasting, the quality of models’ TPDV in the near instrumental era realm under known, but different boundary conditions^54,74^. Although we do not resolve individual El Niño-La Niña events, our record captures the decadal waxing and waning of ENSO such as observed in the instrumental record. Reproduction of ENSO, when compared to instrumental data and where in the surface ocean the signal occurs, is lacking in nearly all of the CMIP5 and CMIP6 models^75^. The lack of accurate reproduction of ENSO in the instrumental era has corollaries to diverse and inconsistent results in future climate change scenario predictions. A better mechanistic understanding of what conditions lead to different expressions of ENSO diversity and how anthropogenic induced climate effects impacts the background climate, including TPDV, is needed to better predict ENSO variability and diversity in the future.

## Methods

Live (K1) and sub-fossil (K2) *K. haumeaae* specimens were collected using DSRV Pisces IV and V from ∼ 400 m depth offshore Kingman Reef (6° 23′ N, 162° 25′ W) in 2005. Skeletons were first washed with seawater and then fresh water before being air-dried on deck. Cross section disks ∼ 0.7 cm thick were cut from close to the basal attachment, polished, and mounted onto glass plates before being drilled by a Merchanteck micromill^45,48,49^. Approximately 2–3 mg of proteinaceous coral skeleton was collected at contiguous 0.1 mm resolution along radial transects from the outer edge to the center. The direct center was avoided due to concerns over the host coral parasitized by the *K. haumeaae* organisms.

Bulk δ^15^N, δ^13^C, and C/N analyses were performed synchronously on ∼ 0.3 mg raw material using a Carlo Erba 1108 elemental analyzer coupled to a ThermoFinningan Delta Plus XP isotope ratio mass spectrometer. Results are reported in conventional per mil (‰) notation relative to air and VPDB standards for δ^15^N and δ^13^C, respectively. Reproducibility based on independent coral replicates is consistent with the 1-sigma sd of the acetanilide standard of < 0.1‰ for both isotopes. Analysis of replicate and standard C/N values indicate a 1-sigma sd reproducibility of 0.2.

Radiocarbon (^14^C) analyses were performed on ~ 1 mg acid-pretreated sub-samples (n = 6 for K1, n = 7 for K2). The calibrated model for each coral was generated in a Bayesian framework^76^. Post-1950 ages in K1 were estimated using a surface water F^14^C reconstruction from nearby Palmyra^77^. The remaining ^14^C ages were calibrated using a local reservoir (ΔR) correction of − 134 ± 25 years^78^ and the Marine20 database^79^. Average age model uncertainties at the ± 95% confidence interval for each coral is approximately ± 110 years.

Utilizing C/N values the outermost 1–2 mm of K2, the subfossil specimen, was determined to be likely impacted by diagenesis and so was excluded from our interpretation^45,49^. Data for these samples are included in the supplement for completeness.

Spectral analysis of the bulk δ^15^N and δ^13^C time-series was independently performed on each record. Each record was first linearly interpolated to their average resolution: K1 to 7 years and K2 to 5 years. The spectral analysis was performed on detrended, z-score normalized, and simple interpolated versions of the records. Final analysis included passing the data through a 3-point (21 year) or 5-point (25 year) moving average filter for K1 and K2, respectively. Since our focus is natural variability, we excised the post 1880 CE portion of K1. We utilized both a Blackman-Tukey method implemented in the ARAND package^80^ where we lagged, by one-third, the auto-covariance function and a Multi-Taper Method as implemented in Kspectra (Spectraworks), using 3 tapers, compared against a red-noise AR (1) process with robust noise estimated from the data-set being analyzed. The Blackman-Tukey method is reported against the 95% confidence interval, and the Multi-Taper Method reported with predicted curves at the 90%, 95%, and 99% confidence interval. Spectral signatures were not dependent on data handling (z-score, detrended, simple interpolation).

Compound specific isotope amino acid (CSIA-AA) δ^15^N analyses were performed utilizing skeletal material from combining adjacent layers to have enough material (~ 6–10 mg) for measurements. Analysis involved established wet chemistry protocols for coral materials (e.g. McMahon et al. ^46^) where amino acids (AAs) were liberated using standard acid hydrolysis conditions (1 ml of 6 N HCl at 110 °C for 20 h), then spiked with a norleucine (Nor-Leu) internal standard and derivatized^81^, followed by purification with cation-exchange chromatography and a salt-removal step (p-buffer = KH_2_PO_4_ + Na_2_HPO_4_ in Milli-Q water, ph 7) before a 3× chloroform rinse and centrifugation before the final conversion to trifluoroacetyl/isopropyl ester derivatives. Derivatized samples were injected in triplicate on a coupled Gas Chromatography-IRMS (ThermoTrace GC, coupled to a Delta + IRMS). Injections were made utilizing concentrations adjusted to produce at least 80 mV IRMS N_2_ signal intensity for the smallest peaks (typically phenylalanine and isoleucine).

Isotope ratios were measured on 13 AAs: alanine (Ala), glycine (Gly), serine (Ser), valine (Val), threonine (Thr), leucine (Leu), isoleucine (Ile), proline (Pro), phenylalanine (Phe), tyrosine (Tyr), lysine (Lys), glutamine + glutamic acid (Glx), and asparagine + aspartic acid (Asx). A range of verification approaches were simultaneously employed to ensure accurate CSIA-AA data. The Nor-Leu internal standard was used to verify that injections gave expected results. Bracketed standards of l-amino acids were repeatedly analyzed after every triplicate coral skeleton injection, and the average measured value of each external standard AA across the entire run was compared against its authentic value, and any systematic bias/offset used to correct sample AA values^82^. Lastly, we utilized an internal laboratory reference material (dried/homogenized cyanobacteria) which was run with each sample batch to evaluate accuracy against a long term (> 10 years) internal control, ensuring long-term reproducibility for individual AA isotope values. Analytical reproducibility from triplicate sample injections was, in general, < 1‰ for δ^15^N CSIA-AA.

From the CSIA-AA data weighted averages of source AAs (Tyr, Lys, and Phe) were calculated using the analytical uncertainty as the weighting function^83^. The presence of heterotrophic microbial resynthesis was estimated using the CSIA-AA metric ∑V^84^.

A total of five (0.5–1 mg) samples (K1 n = 2 and K2 n = 3) from the same CSIA-AA composite samples was analyzed for AA molar concentrations. Wet chemical protocols for AA measurements in proteinaceous corals followed established protocols^48,49^. The AA mole percent (mol%) compositions were quantified using a GC–MS (Agilent 7890 GC coupled to a 5975 MSD) based on single ion monitoring data of the major ion relative to authentic AA external standard calibration curves. Commercial AA standards (Pierce Biochemicals) were used to create concentration series, and response factors from these external standards were used to calculate relative molar concentrations. Reproducibility, as measured by the standard deviation of GC–MS replicates analyses, typically averaged < 5 mol%.

Where possible, records which rely on 14C chronologies and discussed in the manuscript or presented in figures have been updated using 14C-calendar frameworks of IntCal20/SHCal20/Marine20^79,85,86^, and local estimates of ∆R (www.calib.org). Without the individual varve data, we were unable to update the Laguna Palcacocha ENSO reconstruction^61^. We do note that the discrete 14C data used as scaffolding by Moy et al., when calibrated using SHCal20, are less than 100 calibrated years different than the (IntCal98) calibrated ages presented in Rodbell for the same core^87^. The El Junco sand record^60^, an ENSO proxy, has an age model uncertainty which waxes and wanes from ± 10 years (where 210Pb constrains the upper portion) to ± 195 years, and averages 115 years (95% CI). Spectral comparison with western North America tree-ring based PDSI^20,21^ was done for discrete sections of the tree-ring reconstruction consistent with the age spans of the two *Kulamanamana* specimens and including the 95% CI uncertainty in the coral age-models. Annual tree-ring data were filtered with a 21 year moving-average filter prior to spectral analysis via convolution of the 1/3-lagged autocovariance function, or via the Multi-Taper Method.

We note that in the literature there are more records to compare our data against, including paleosalinity and precipitation proxy records from the Makassar Straits^63,88,89^. These sediment cores’ data, except where constrained by 210Pb, when put into a Bayesian age-model framework, have an approximately ± 200 year age model uncertainty, or twice that of ours. Such uncertainty in the age-model would lead to a less than ideal comparison. We note that these paleosalinity data were differenced against a Galapagos precipitation sand-based proxy record^60^ to estimate past variations in the Southern Oscillation Index^90^. Yan et al. only included the analytical uncertainty of the underlying 14C ages, not the calibrated age uncertainties: i.e., the temporal uncertainty is underestimated by a factor of 2–3×, which leads to fundamental questions in the SOI reconstruction itself. Within the framework of documenting the cyclicity in the CEP δ^15^N data and ascertaining if it is consistent with modern TPDV or not, comparison with all possible paleo-records is not required.

### Supplementary Information

Supplementary Information 1.

Supplementary Information 2.

## Data Availability

In addition to being provided as a Supplementary Datafile, these data are digitally archived at NCEI/NOAA Paleoceanography (https://www.ncei.noaa.gov/access/paleo-search/study/38820).
