# Supplementary material for: Tropical decadal variability in nutrient supply and phytoplankton community in the Central Equatorial Pacific during the late Holocene
Source: Sci Rep. 2024 Feb 20;14:4161. doi: 10.1038/s41598-024-54635-3 (PMC10879156; doi:10.1038/s41598-024-54635-3)
Supplement: Supplementary file 2 — Supplementary Information 2. [file 41598_2024_54635_MOESM2_ESM.pdf]

Tropical Pacific Decadal Variability in nutrient supply and phytoplankton community in the Central Equatorial Pacific during the late Holocene.

T. P. Guilderson, D. S. Glynn, and M. D. McCarthy
Ocean Sciences Department
University of California – Santa Cruz

Supplementary File

Supplementary Table 1. Least squares correlation analysis of skeletal bulk and CSIA-AA $\delta^{15}N$. A Monte Carlo simulation was used to estimate the 95% confidence interval (CI) of the slope. The analysis was done for groupings of the analyzed source AAs: phenylalanine (Phe), lysine (Lys), and tyrosine (Tyr). *Kulamanamana haumeaae* data are from the Line Islands (this study), a live-collected specimen from Cross Seamount (Glynn et al., 2019), and live-collected and sub-fossil specimens from near Oahu (Sherwood et al., 2014). The analysis used the weighted mean of the source AA $\delta^{15}N$ values using the analytical uncertainty as the weighting factor and the one-sigma standard deviation reproducibility of $\delta^{15}N_{bulk}$. All the relationships at the 95% CI have a slope close to 1 with source AA and bulk sharing more than 80% of the variance. See Supplementary Figure 02 for a representative plot of the data.

Source AAs	Intercept	SLOPE	95%CI	R-squared
Phe + Lys	-5.74	1.08	.99-1.17	0.88
Tyr + Lys	-2.32	0.86	.83-.89	0.96
Tyr + Phe	-4	0.89	.82-.97	0.83
Tyr + Lys + Phe	-2.84	0.85	.77-.93	0.92

Supplementary Table 2. Type II correlation analysis of $\delta^{15}N$ and $\delta^{13}C$ bulk isotope data of Line Island *Kulamanamana haumeaae (this study)*. Analysis included the one-sigma standard deviation reproducibility of bulk skeletal $\delta^{15}N$ and $\delta^{13}C$.

Time Frame	R-squared
13-2005 (all data)	0.38
13-850 (specimen K2)	0.65
1232-2005 (specimen K1)	0.55
1232-1850 (K1, no industrial era)	0.12

Supplementary Table 3. Multi-taper method, general peaks and confidence intervals. We note that regardless of pre-analysis handling: none, simple moving average, triangle-filter, detrending, and Z-score normalization that the spectra across the analyses were consistent with each other. Moreover, the significant peaks are consistent with the results of the Blackman-Tukey analysis. Results are shown for simple moving averages as described in the methods section. Note the consistent ˜50-60 year periodicity in both the $\delta^{15}N$ (water-mass) and $\delta^{13}C$ (phytoplankton community) data.

	**Frequency**	**Period**	**CI%**
Kingman 2 $\delta^{15}N$			
	0.0092	109	99
	0.0182	55	99
	0.0551	18	90
Kingman 1 $\delta^{15}N$			
	0.0054	184	95
	0.0163	61	99
	0.0294	34	99
Kingman 2 $\delta^{13}C$			
	0.0180	56	99
Kingman 1 $\delta^{13}C$			
	0.0091	110	99
	0.0163	61	99
	0.0233	43	95

Supplementary Figure 01. Annual mean surface Nitrate [NO_3] concentration. Contours are at the 1 µmole-kg^{-1} interval. Kingman Reef is demarked by the star. Data are from the World Ocean Atlas, 2018 (Garcia et al., 2018).

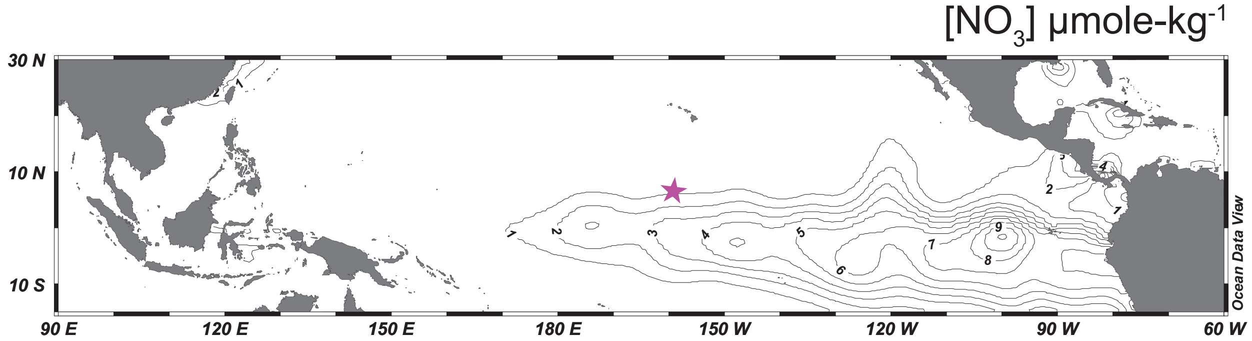

Supplementary Figure 02 Bulk skeletal $\delta^{15}N$ and source AA $\delta^{15}N$, (‰) relative to air. The source AA average includes Tyrosine (Tyr), Phenylalanine (Phe), and Lysine (Lys) and uses the analytical 1-sigma standard deviation as the weighting function.

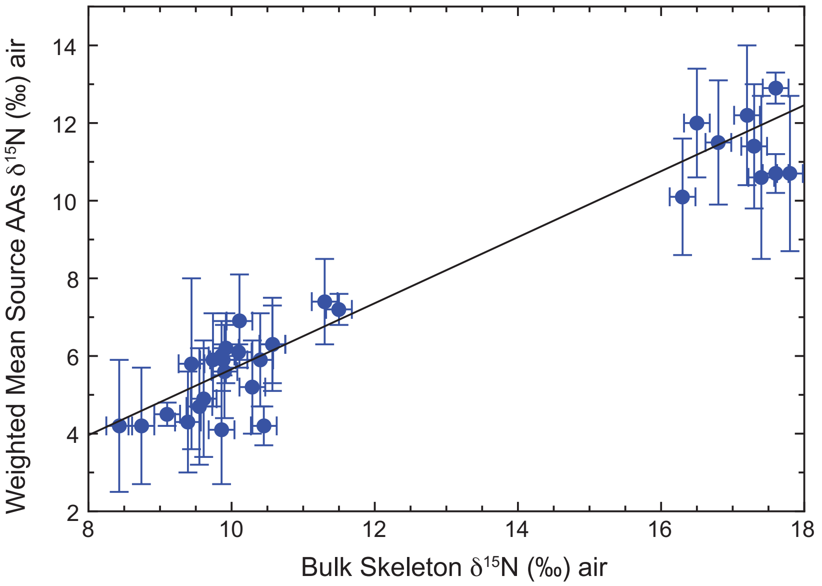

Supplementary Figure 03. Summary of *Kulamanamana haumeaae* amino acid relative mole percentage. Note, the methodology used in these studies does not afford the recovery of histidine, which can be 30% of the total amino acid content (Goodfriend 1997). Makapuu (Oahu) data are from Sherwood et al., (2014), Cross Seamount data are from Glynn et al., 2022, and Kingman Reef (this study). The Cross Seamount ancient sub-fossil specimen's outer skeleton was visibly weathered. Each specimens' data have been averaged for clarity. Individual AA mol% estimates have ±5% uncertainties. The mole% data indicate good preservation of the Kingman Reef, Line Islands, samples.

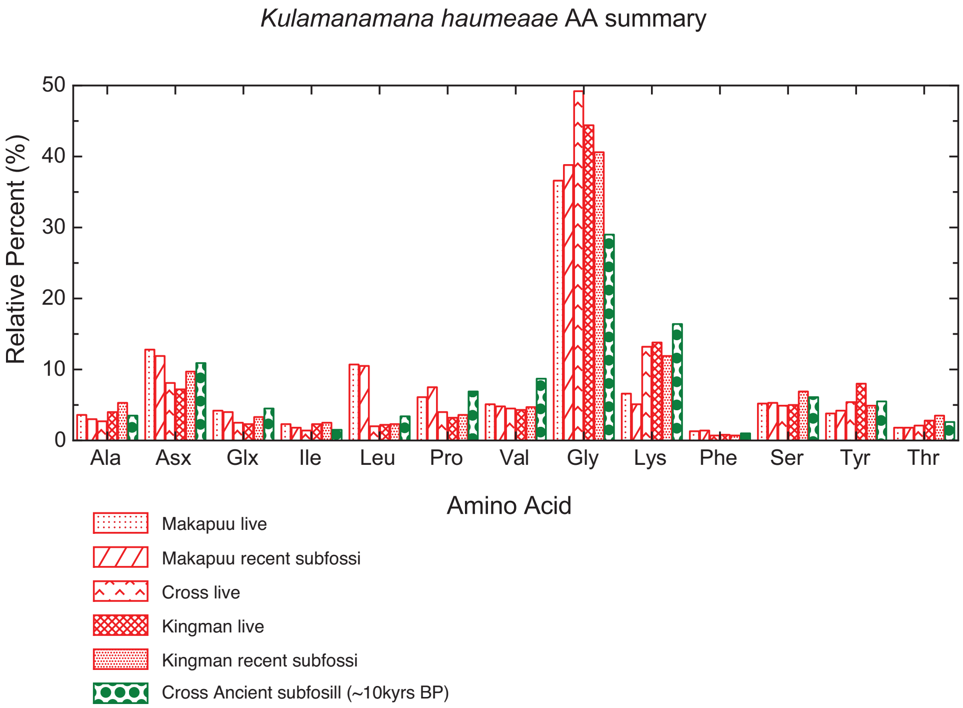

Supplementary Figure 04. Line Island *Kulamanamana haumeaae* skeletal bulk $\delta^{15}N$ and $\delta^{13}C$. Specimen K1 (1230-2005 CE) is presented in blue, with post-LIA and the industrial era (1880-2005 CE) as open blue symbols. Specimen K2 (13-854 CE) is presented as orange.

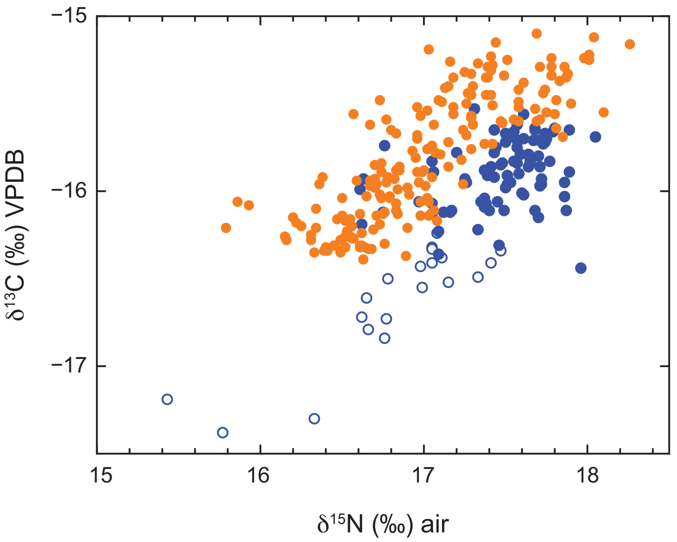

Supplementary Figure 05. Representative sediment reconstructions of export production $\delta^{15}N$ at the origins of the South Equatorial Current (A), and the North Equatorial Current (B). MC 12H (3.8°S 81.1°W 378m water depth) data are from Sauthoff 2016 and CDH23 (3.8°S, 81.1°W, 373m water depth) are from Bova et al., 2018. MD982181 (6.2°N, 125.5°E, 2114m water depth) data are from Kienast et al., 2008, using the radiocarbon data of Stott et al., (2004). The average age-model 95% CI uncertainty interval for MC 12H is ±175 years, CDH 23 ±240 years, and ±205 years for MD98 2181.

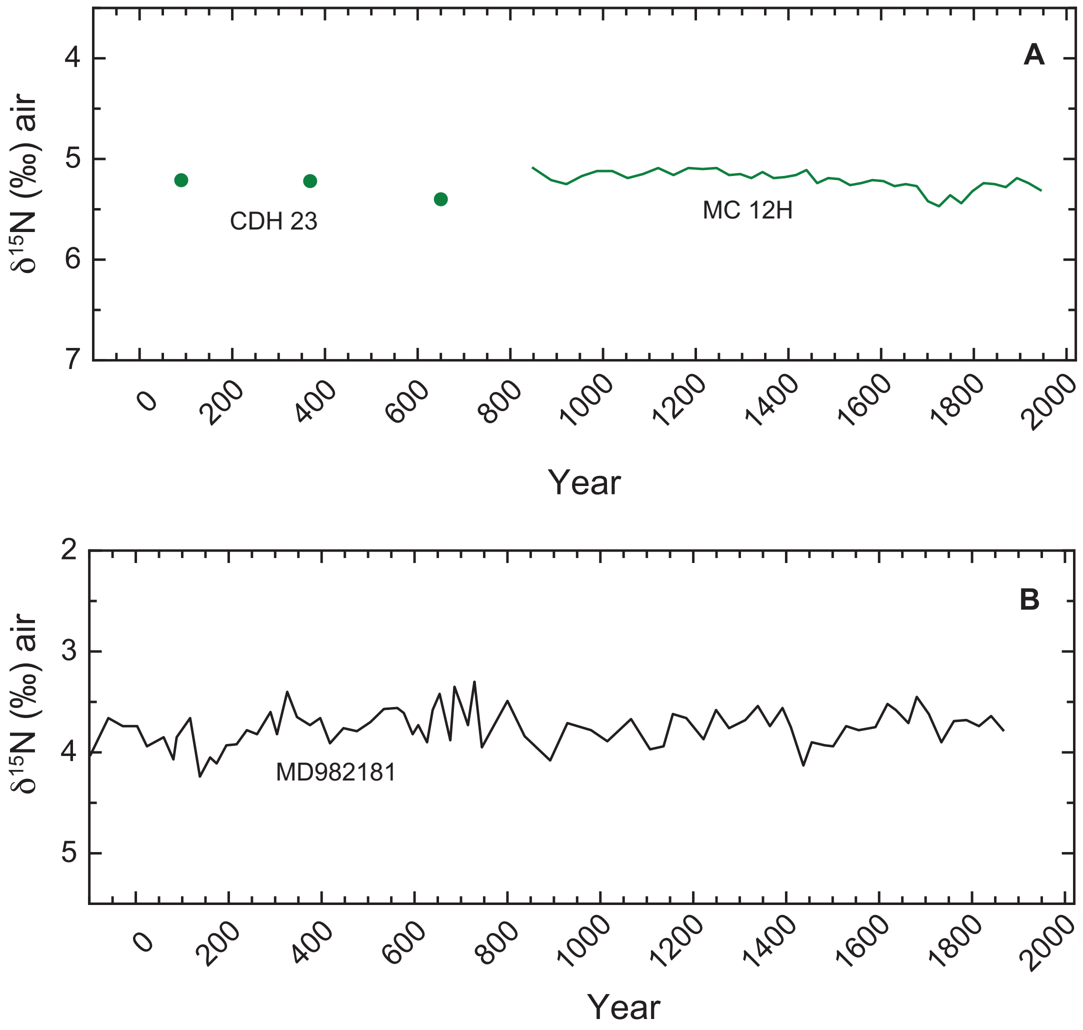

Supplementary Figure 06. δ^{15}N variability in the far western Pacific from near Mindanao (MD982181) and Palau (*Muricella*), in the path of the NECC. The MD982181 data are conventional sediment bulk δ^{15}N (Kienast et al., 2008), as shown in Supplementary Figure 05b. The Palau (7.3°N, 134.5°E) data are from a black coral, *Muricella*, collected from near the base of the nutricline (Williams and Grottoli, 2010a; Williams et al., 2016). *Muricella* primarily feed upon suspended particulate organic carbon (Williams and Grottoli 2010b). Thus, the *Muricella* data records mixed layer baseline nitrate variability but potentially slightly biased by in situ (at depth) phytoplankton photosynthesis. Both data sets have been differenced to their respective means. Note the decrease in the Palau δ^{15}N that is accentuated in the latter half of the 20th century.

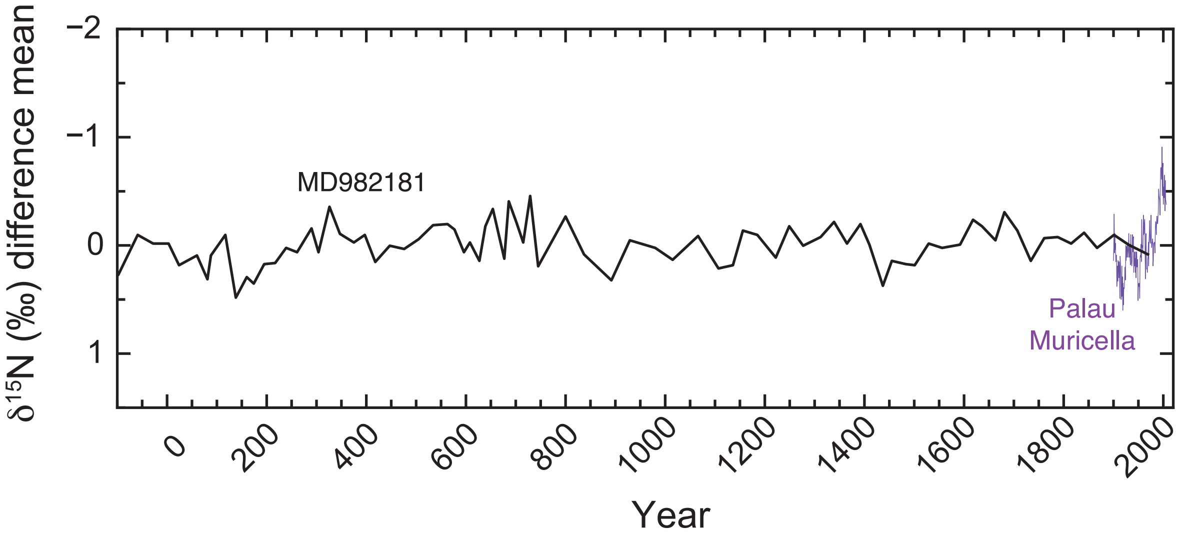

Supplementary Figure 07: Multi-decadal variability (TPDV) of the CEP ($\delta^{15}N$, blue line) and the Palmer Drought Severity Index (PDSI, orange line) as reconstructed by Cook et al., (2004; 2010) for the American West (33-43°N, 106-119°W). The $\delta^{15}N$ data from K2 was first linearly detrended to create a residual (or difference from the mean) and then Z-score transformed. The $\delta^{15}N$ data from K1, which has no secular long-term trend prior to the Industrial era, was differenced from the mean and Z-score transformed. There is no significant difference in the Z-score results as a function of handling these two records independently or after normalization to the mean, or comingling the two data sets prior to Z-score normalization. The PDSI data, which is annual in nature, have been passed through a 21year moving average filter. We posit, given the coral's age-model uncertainty, that the high TPDV period recorded near ~1400 is most likely time equivalent to the ~1350 wet period with the corresponding low TPDV in the coral coincident with the dry period in the mid 1200s. Similar within age-model uncertainties alignment could be done for the earlier portion of the CEP record. That our record is not an exact 1:1 match with the American SW PDSI is not unexpected. Firstly, baseline nitrate is not temperature. Moreover, using ENSO as a 'model' for describing interannual variability, including that forced through teleconnections, there is not always a direct match between eg., the Niño3.4 index and another climate record that is influenced by ENSO. In this case, more than just TPDV and ENSO influences precipitation in the American southwest.

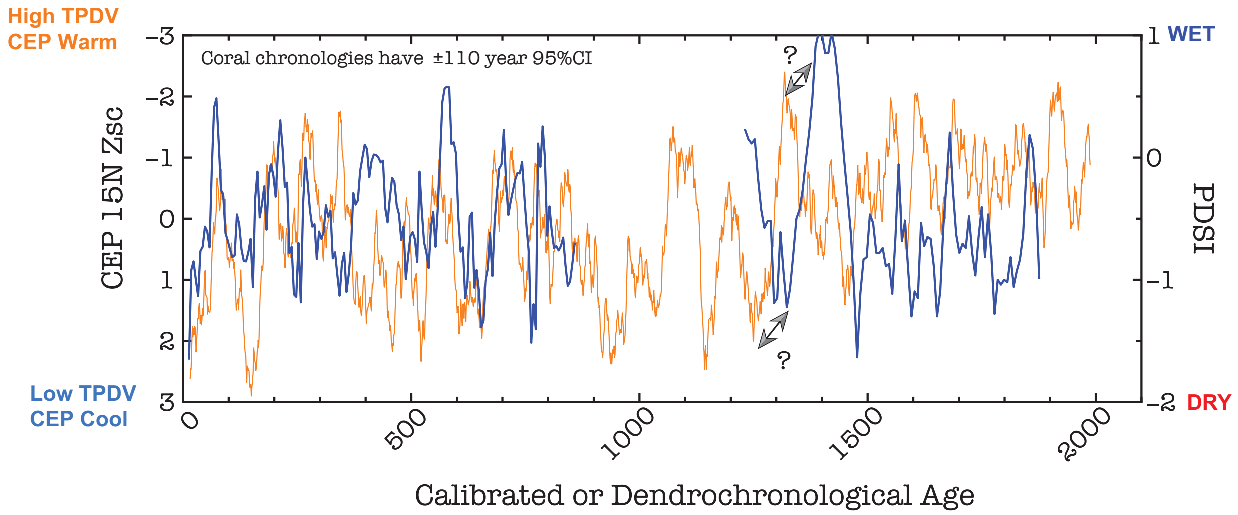

Supplementary Figure 08: MTM spectral analysis results of the US American West PDSI and Blackman-Tukey comparison of CEP $\delta^{15}N$ and PDSI spectral character. A) PDSI over the time shared with K1 (1220-1880 CE) B) PDSI over the time shared with K2 (13-858 CE) and C) Individual Blackman-Tukey spectral results of the PDSI and K1 and K2. Palmer Drought Severity Index (PDSI) for the North American west (33-43°N, 106-119°W: data of Cook et al., 2004 and 2010). MTM results are compared against 90, 95, and 99% confidence intervals using robust red-noise and the Blackman-Tukey results against the 95% confidence interval. Note both the CEP $\delta^{15}N$ and PDSI share similar 50-60 year variability.

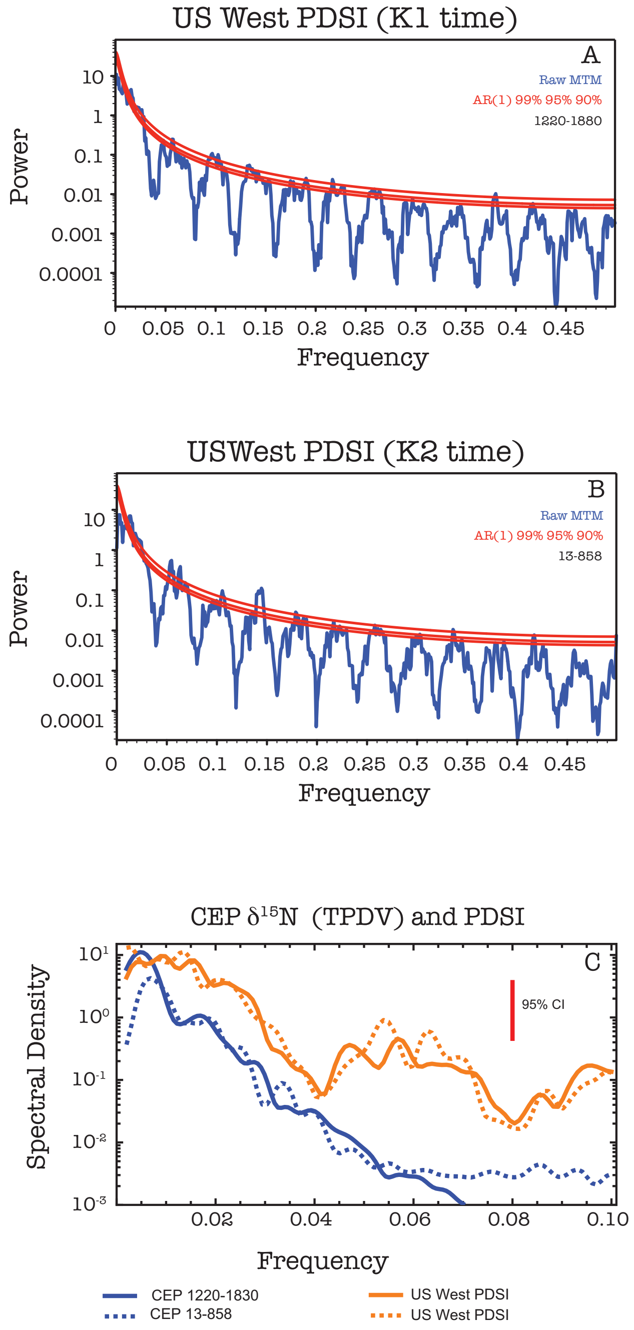

Supplementary Figure 09: Spectral analysis of the Kingman Reef δ^{13}C data. A) Multi-taper method results of the K1 specimen (1220-1880 CE) B) Multi-taper method results of the K2 specimen (13-858 CE) and C) Blackman-Tukey results of K1(solid, bold line) and K2 (dashed line), together. Confidence significance levels for the MTM results were determined using robust red-noise intrinsic to the data. The 95% confidence interval is shown for the Blackman-Tukey results. Data were handled as described in the methods section.

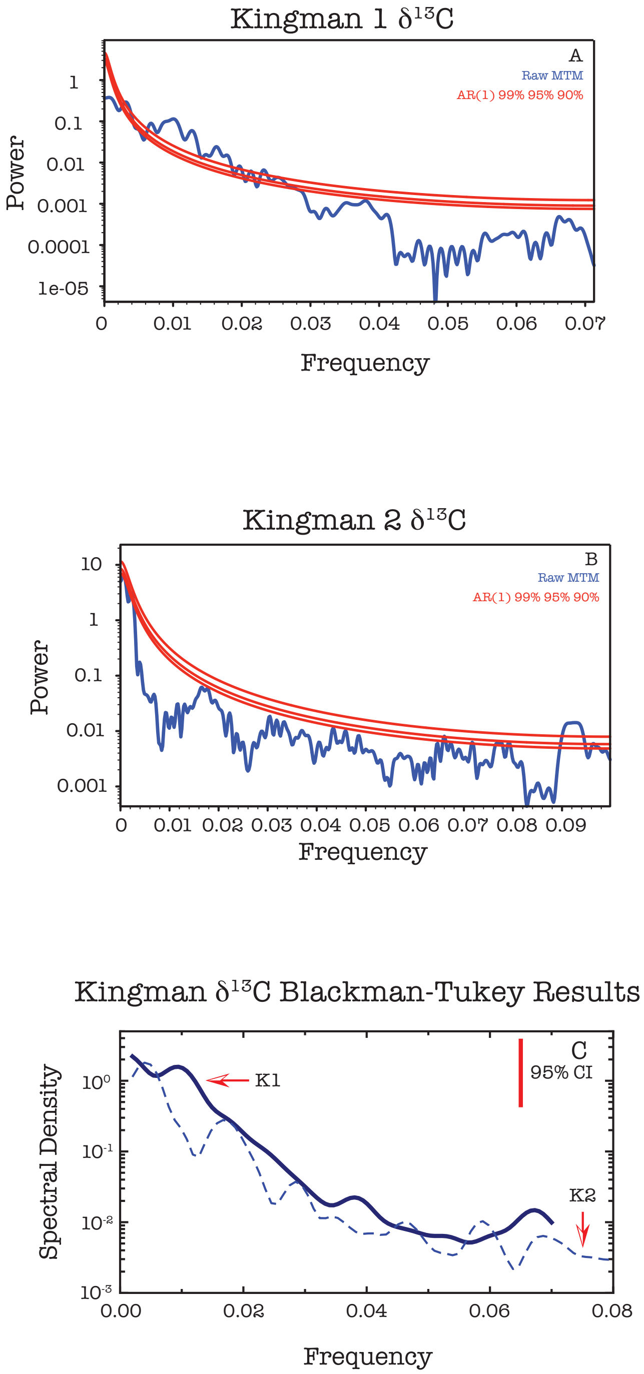

Supplementary File References

Bova, S.C., Herbert, T.D., and Altabet M.A. Ventilation of northern and southern sources of aged carbon in the eastern equatorial Pacific during the Younger Dryas rise in atmospheric CO2. *Paleoceanography and Paleoclimatology, **33**, 1151-1168. doi: 10.1029/2018PA003386* (2018).

Cook, E. R. *et al.* Megadroughts in North America: placing IPCC projections of hydroclimatic change in a long-term palaeoclimate context: Megadroughts in North America. *J. Quaternary Sci.* **25**, 48–61 (2010).

Cook, E. R., Woodhouse, C. A., Eakin, C. M., Meko, D. M. & Stahle, D. W. Long-Term Aridity Changes in the Western United States. *Science* **306**, 1015–1018 (2004).

Garcia, H. E., *et al.* World Ocean Atlas 2018, Volume 4: Dissolved Inorganic Nutrients (phosphate, nitrate and nitrate+nitrite, silicate). A. Mishonov Technical Ed.; NOAA Atlas NESDIS 84, 35pp. (2018).

Glynn, D. S., McMahon, K. W., Sherwood, O. A., Guilderson, T. P., and McCarthy, M. D. Investigating preservation of stable isotope ratios in subfossil deep-sea proteinaceous coral skeletons as paleo-records of biogeochemical information over multimillennial timescales. *Geochimica et Cosmochimica Acta, **338**, 264-277* (2022).

Goodfriend, G. A. Aspartic acid racemization and amino acid composition of the organic endoskeleton of the deep-water colonial anemone Gerardia: Determination of longevity from kinetic experiments. *Geochimica et Cosmochimica Acta.*, **61**, 1931-1939 (1997).

Kienast, M., Lehmann, M. F., Timmermann, A., Galbraith, E., Bolliet, T., Holbourn, A., Normandeau, C., and Laj, C., 2008. A mid-Holocene transition in nitrogen dynamics of the western equatorial Pacific: Evidence of a deepening thermocline? *Geophysical Research Letters., **35**, L23610, doi: 10.1029/2008GL035464.*

Sauthoff, W. Nitrogen isotopes of amino acids in marine sediment: A burgeoning tool to assess organic matter quality and changes in supplied nitrate d15N. Masters Thesis. University of California, Santa Cruz, CA 91pp. (2016).

Sherwood, O.A., Guilderson, T. P., Batista, F. C., Schiff, J. T., and McCarthy, M. D. Increasing subtropical North Pacific Ocean nitrogen fixation since the Little Ice Age. *Nature, **505**, 78-81, doi:10.1038/nature12784* (2014).

Stott, L.D., K.G. Cannariato, R.C. Thunell, G.H. Haug, A. Koutavas, and S.P. Lund. Decline of surface temperature and salinity in the western tropical Pacific Ocean in the Holocene epoch*. Nature, v.* ***431****, p. 56-59* (2004).

Williams, B., and Grottoli, A. G. Recent shoaling of the nutricline and thermocline in the western tropical Pacific. *Geophysical Research Letters,* ***37****: L22601 doi:10.1029/2010GL044867.* (2010a).

Williams, B., and Grottoli, A. G. Variability in $\delta^{15}N$ and $\delta^{13}C$ values of alcyonacean and antipatharian corals from the western equatorial Pacific. *Geochimica et Cosmochimica Acta* ***74****: 5280–5288. doi:10.1016/j.gca.2010.06.026* (2010b).

Williams, B., Thibodeau, B., Chikaraishi, Y., Ohkouchi, N., Walnum, A., Grottoli, A. G., and Colin, P. L. Consistency in coral skeletal amino acid composition offshore of Palau in the western Pacific warm pool indicates no impact of decadal variability in nitricline depth on primary productivity. *Limnology and Oceanography,* ***62,*** *399-407* (2017).
